# Dynamic QT response to cold‐water face immersion in long‐QT syndrome type 3

**DOI:** 10.1111/ped.14319

**Published:** 2020-08-06

**Authors:** Kazuhiro Takahashi, Wataru Shimizu, Naomasa Makita, Mami Nakayashiro

**Affiliations:** ^1^ Department of Pediatric Cardiology Okinawa Nanbu and Children’s Medical Center Okinawa Japan; ^2^ Division of Cardiology Nippon Medical School Tokyo Japan; ^3^ National Cerebral and Cardiovascular Center Research Institute Osaka Japan

**Keywords:** arrhythmia, face immersion, long‐QT syndrome type 3, QT dynamics, ventricular repolarization

## Abstract

**Background:**

Abnormal dynamics of QT intervals in response to sympathetic nervous system stimulation are used to diagnose long‐QT syndrome (LQTS). We hypothesized that parasympathetic stimulation with cold‐water face immersion following exercise would influence QT dynamics in patients with LQTS type 3 (LQT3).

**Methods:**

Study participants (*n* = 42; mean age = 11.2 years) comprised 20 genotyped LQTS children and 22 healthy children. The LQTS group was divided into LQT3 (*n* = 12) and non‐LQT3 (*n* = 8) subgroups. Provocative testing for assessing QT dynamics comprised a treadmill exercise followed by cold‐water face immersion. The QT intervals were automatically measured at rest and during exercise, recovery, and cold‐water face immersion. The QT/heart rate (HR) relationship was visualized by plotting beat‐to‐beat confluence of the data.

**Results:**

The QT/HR slopes, determined by linear regression analysis, were steeper in the LQTS group than in the control group during exercise and immersion tests: −2.16 ± 0.63 versus −1.21 ± 0.28, *P* < 0.0001, and −2.02 ± 0.76 vs −0.75 ± 0.24, *P* < 0.0001, respectively. The LQT3 patients had steeper slopes in the immersion test than did non‐LQT3 and control individuals: −2.42 ± 0.52 vs −1.40 ± 0.65, *P* < 0.0001, and vs −0.75 ± 0.24, *P* < 0.0001.

**Conclusions:**

The QT dynamics of LQT3 patients differ from those of other LQTS subtypes during the post‐exercise cold‐water face immersion test in this study. Abnormal QT dynamics during the parasympathetic provocative test are concordant with the fact that cardiac events occur when HRs are lower or during sleep in LQT3 patients.

Congenital long‐QT syndrome (LQTS) is an inherited channelopathy characterized by prolonged myocardial repolarization, conferring an increased predisposition for polymorphic ventricular arrhythmias and risk of sudden cardiac death.^1^ The most common subtypes are caused by mutations in the genes *KCNQ1* (mutation of which causes LQTS subtype 1, LQT1), *KCNH2* (LQT2), and *SCN5A* (LQT3).^2^, ^3^, ^4^ The diagnostic probability of LQTS is high when the corrected QT (QTc) interval at rest exceeds 480 ms. However, a recent article clearly defined the difficulties in diagnosing LQTS using only the baseline electrocardiogram.^5^

Epinephrine or exercise stress testing are used as provocative test modalities for the diagnosis of LQTS, in particular, for LQT1 or 2.^6^, ^7^, ^8^, ^9^, ^10^ These tests are based on the paradoxical prolongation or abnormal dynamics of QT intervals in response to stimulation of the sympathetic nervous system, which is pathognomonic for LQT1 and 2.^11^, ^12^

The subtypes of LQTS, including LQT3, can be concealed diseases.^13^, ^14^ Furthermore, LQT3 shows little response to sympathetic stimulation; thus, the epinephrine stress test may fail to identify patients with LQT3 because of a supernormal QT shortening in response to sympathetic nervous system stimulation.^13^, ^15^ Several reports have suggested that LQT3 can be suspected following the exclusion of LQT1 and LQT2 by the epinephrine bolus test. The lack of information available regarding the QT behavior/dynamics of LQT3 hinders definitive conclusions about the condition.^13^, ^16^, ^17^, ^18^

We have previously reported that the QT dynamics (QT/heart rate slope) of healthy children can be evaluated using a new multifunctional electrocardiogram (ECG) recorder, Radarcirc™ (Nihon Kohden Co. Ltd, Tokyo, Japan).^19^ Individuals with LQT3 or other LQTS subtypes have abnormal QT dynamics during exercise stress testing, which can be identified using this equipment. However, this method is unable to differentiate LQT3 from the other subtypes.^20^

We hypothesized that stimulation of the parasympathetic nervous system with exercise followed by cold‐water face immersion (CWFI) augments parasympathetic re‐stimulation and induces a differential QT response to heart rate (HR) changes in patients with LQT3 compared to patients with other LQTS subtypes. In the present study, we examined this response via the fully automated measurement of QT intervals with a multifunctional ECG recorder. The present study focused on patients with LQT3 caused by the E1784K mutation in *SCN5A*, which is prevalent in the Okinawa island population of Japan.^21^ The aims of this study were therefore as follows: (i) to assess the feasibility of the measurement of QT dynamics during CWFI testing in patients with LQTS; (ii) to characterize QT dynamics during post‐exercise CWFI testing in patients with LQT3.

## Methods

### Study cohort

The study population included children identified in a school‐based ECG screening program for cardiovascular disease, who had been referred to Okinawa Children’s Medical Center (Okinawa, Japan) between April 2010 and January 2016.

The participants included 42 children, comprising 20 asymptomatic children with genotyped LQTS (mean age, 11.1 years; 11 female children) and 22 otherwise healthy children referred for the evaluation of premature ventricular contractions (mean age, 11.9 years; 14 female children). All participants were to be subjected to exercise and CWFI testing. Symptomatic patients and patients on beta‐blocker therapy were not included. Children with wide QRS intervals, bigeminal rhythm, family history of sudden cardiac death, or suspected long‐QT syndrome were excluded from the control group. No child had any evidence of cardiovascular disease on physical examination, chest X‐ray, or echocardiography.

### Study design

This retrospective review was approved by the Institutional Review Board at Okinawa Children’s Medical Center. Written informed consent was obtained from all guardians of the study participants. Data were extracted from the medical records of Okinawa Children’s Medical Center. The assessment of LQTS involved a historic review, including a family history, physical examination, resting ECG, and LQTS genetic screening. Patients who provided informed consent underwent genetic analysis at either the National Cerebral and Cardiovascular Center (NCVC; Osaka, Japan), as outlined previously,^20^ or at Nagasaki University graduate school.

### Electrocardiographic measurements

#### QT interval at rest

Standard 12‐lead ECG recordings were obtained at rest using a six‐channel recorder (model ECG‐8300, Nihon Kohden Co. Ltd) at a paper speed of 25 mm/s, with a calibration of 10 mm/mV. All ECG recordings before exercise testing were performed with the participants in the supine position. One experienced observer (K.T.) manually measured the QT interval in lead V5, using a tangential method with a digitizer. Moreover, all ECG recordings were blinded with respect to the automated measurements. The QT interval was measured from the earliest onset of the QRS complex to the T‐wave offset, defined as the point at which the isoelectric baseline intersected a tangential line drawn at the maximal downslope of the positive T‐wave. The QT and preceding RR intervals were measured for three consecutive beats in lead V5. The average of the three measurements was calculated for precision, and the QT intervals were corrected for HR using Fridericia’s formula. The original Schwartz–Moss scores – derived from the QTc, symptoms, and family history – were ascertained in all LQTS patients.^22^, ^23^

#### Automated ECG measurements during the tests

During the testing, 12‐lead ECG monitoring was conducted using a Radarcirc™ multifunctional ECG recorder. The QT interval was automatically calculated beat to beat. The end of the T‐wave was identified by the tangential method (slope‐intercept method), as described elsewhere.^19^ Measurements made using the ECG recorder were not subjected to manual modification. The QT/HR dynamics were visualized by plotting the beat‐to‐beat confluence of the data offline on a personal computer, after the exclusion of wide QRS complexes. Linear regression analysis was used to ensure that non‐uniform data were represented. We assessed the QT/HR slope according to three phases: during exercise (Ex), during recovery from exercise (Rec), and during CWFI testing (FI). The QT/HR slope relationship was determined by linear regression analysis. Thus, the slope and intercept of the linear equation were obtained. Estimated QT intervals at an HR of 60 beats/min (QT60) were calculated using the regression line formula.

### Exercise stress testing

The exercise stress test was performed as symptom‐limited treadmill testing, using a modified Bruce protocol. Blood pressure, HR, and symptoms were monitored during exercise, a 1 min cooling down period, and a 5 min recovery period. The ECG recordings were obtained on a 6‐channel recorder (Marquette Electronics, Inc., Milwaukee, WI, USA) and on the automated ECG recorder.

### Cold‐water face immersion testing

The CWFI test was performed after 6 min of recovery following the exercise test, using a method published by Yoshinaga *et al.* ^24^ The children were made to sit with their faces immersed in cold water at a temperature of 10 °C while holding their breath for as long as possible after maximum inspiration. Continuous ECG recordings were obtained during this test, and all ECG measurements during CWFI were used for analysis.

### Statistical analysis

Participants’ characteristics are reported as the mean ± standard deviation or median and interquartile range (25th, 75th percentile) for continuous data, and as numbers with percentages for nominal data. Normally distributed continuous variables were compared using the Student’s *t*‐test or analysis of variance (ANOVA). Continuous variables that were not normally distributed were evaluated using the Mann–Whitney *U*‐test for two‐group comparisons (between the control and LQTS groups or between LQT3 and non‐LQT3 groups) and the Kruskal–Wallis test for multiple‐group comparisons. The χ^2^ test was used to compare proportions. The nonparametric Fisher’s test was used to compare proportions that were not suitable for χ^2^ testing. The *P*‐values for multiple comparisons were determined by a two‐way ANOVA followed by Tukey’s post‐hoc test to compare differences between groups. Simple linear regression analyses were performed, calculating the Pearson’s correlation coefficients, regression‐line intercepts, and regression‐line slope to evaluate the relationships between the QT interval and HR and QTc. Two‐tailed *P*‐values < 0.05 were considered statistically significant. Data analysis was performed using JMP version 10.0.1 (JMP, Cary, NC, USA).

## Results

### Study population

Demographic characteristics of the patients and controls are summarized in Table 1. The participants comprised 17 male and 25 female children, with a mean age of 11.2 ± 3.4 years (range, 6–19 years). The control group comprised 22 unrelated healthy children without QT prolongation (mean age, 11.9 years; 14 female children) and the LQTS group comprised 20 genotyped LQTS patients: LQT3 (*n* = 12), LQT1 (*n* = 3), LQT2 (*n* = 3), LQT7 (*n* = 1), and LQT8 (*n* = 1; genetic details are in Table S1). The LQTS patients were divided into two subgroups: LQT3 (*n* = 12) and non‐LQT3 (*n* = 8) subgroups. The proportions of female patients was similar in the control and LQTS groups. However, female children represented a higher proportion of the non‐LQT3 subgroup than the LQT3 subgroup. Electrocardiogram parameters, such as resting HRs and QTc intervals, differed between the controls and all LQTS group / subgroups. The QT and QTc intervals did not differ significantly between sexes in any group / subgroup (data not shown). All exercise testing parameters, such as peak HR, percentage target HR, and double products (double product = maximal HR × systolic blood pressure), were smaller in the LQTS group than in the controls. The QTc intervals at 4 min of recovery after exercise – an added criterion of the revised Schwartz–Moss score – were also compared between the groups. The QTc intervals were significantly longer in the non‐LQT3 subgroup than in the control group (*P* < 0.01) but showed no significant difference between the LQT3 subgroup and control group (*P *> 0.05). There were no significant arrhythmias (including premature ventricular contractions) during CWFI, with the exception of isolated premature atrial contractions.

**Table 1:** Clinical, ECG, and exercise testing / cold water face immersion characteristics

	Control	LQTS	Subtypes	
		Total	Type 3	Non‐type 3
	n = 22	n = 20	n = 12	n = 8
Female sex (%)	14 (64%)	11 (55%)	4 (33%)^**^	7 (78%)
Age, years	13.0 (8.8, 14.3)	9.5 (7.3, 12.3)	9.5 (7.3, 12.0)	10.5(7.3, 13.8)
Height, cm	148 ± 15	139 ± 20	138 ± 17	140 ± 24
Weight, kg	43 ± 12	36 ± 15*	34 ± 11	38 ± 20
Schwartz score	0	3.3 ± 1.1*	3.3 ± 1.0	3.4 ± 1.3
Resting ECG				
HR, bpm	73 ± 11	64 ± 8**	61 ± 8	67 ± 8
QRS, ms	87.5 ± 9.9	84.7 ± 12.0	89.0 ± 11.4	78.3 ± 10.4
QTc (Fridericia), ms	413 ± 18	510 ± 43**	518 ± 45	498 ± 38
Exercise testing				
Peak HR, bpm	174 ± 12	155 ± 21*	163 ± 12^*^	144 ± 26
% Target HR	84 ± 6	74 ± 10*	78 ± 6^*^	70 ± 14
Double product	24,691 ± 5,035	19,176 ± 4,337*	19,631 ± 2,768	18,494 ± 6,176
QTc at 4 min after maximal effort	432 ± 29	497 ± 48**	474 ± 14^*^	532 ± 60
Cold water face immersion				
Duration, s	30 (27, 35)	31 (26, 35)	31 (26, 35)	32 (26, 35)
Minimal HR, bpm	52 (47, 56)	51 (47, 53)	51 (47, 53)	51 (41, 58)
Ratio of minimal/maximal HR	0.47 (0.43, 0.52)	0.49 (0.45, 0.51)	0.49 (0.46, 0.51)	0.47 (0.43, 0.52)

*P < 0.05, **P < 0.01. Double product = maximal heart rate × systolic blood pressure. Values are means ± SD or median (interquartile range, IQR) where indicated. ECG, electrocardiogram; HR, heart rate; QTc, corrected QT interval; LQTS, long QT syndrome.

### Dynamic QT responses to HR changes in exercise and cold‐water face immersion

**Fig. 1:**
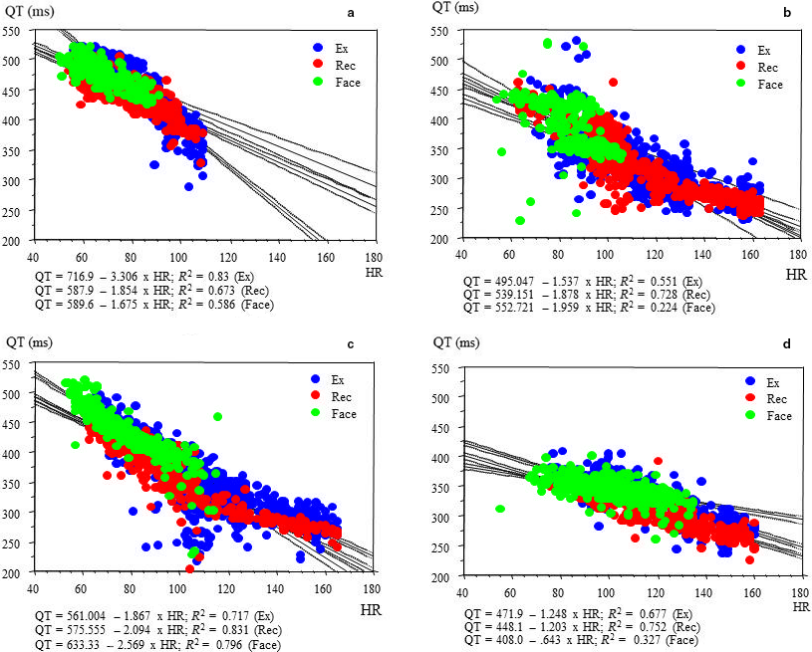
Differential QT dynamics during exercise and face immersion (a) LQT 1 (Case 14); (b) LQT2 (Case 16); (c) LQT 3 (Case 9); (d) control (Case 39). Blue, red and green dots indicate QT intervals during exercise, recovery and face immersion, respectively.

**Fig. 2:**
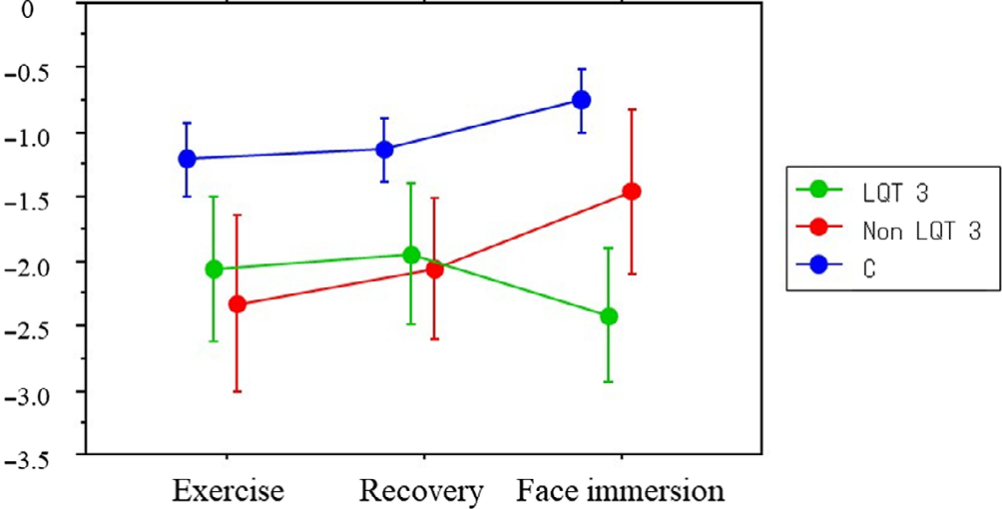
QT/HR slope during exercise and face immersion Blue, red and green dots indicate control, non‐LQT3 and LQT3, respectively.

**Fig. 3:**
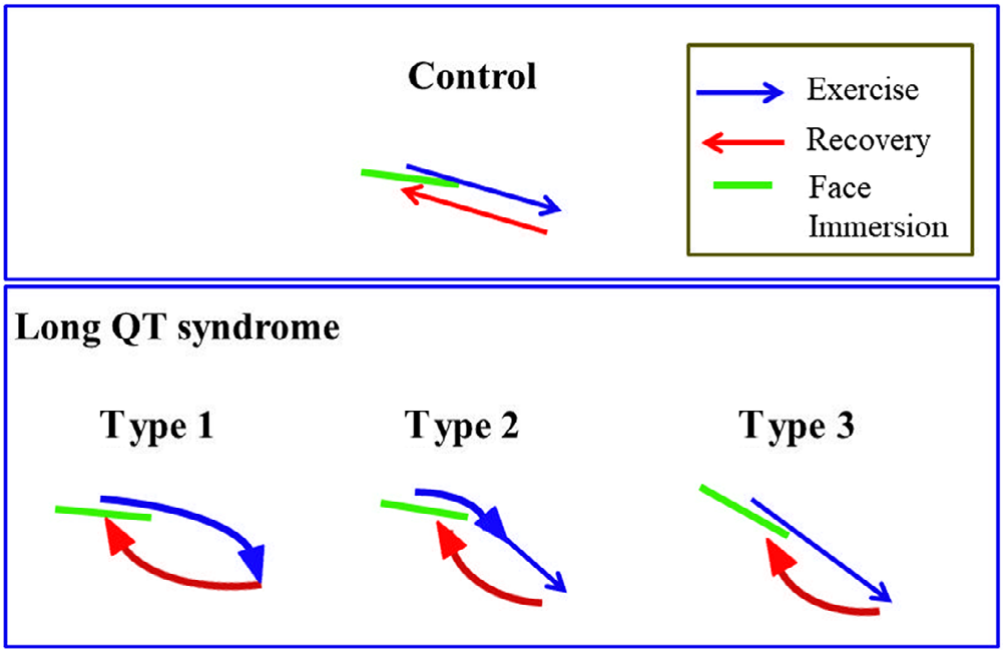
Schematic representation of differential QT/ HR relationship during exercise and face immersion.

The slope and intercept of the linear equation and QT60 for each phase of exercise testing (exercise and recovery) and post‐exercise CWFI are summarized in Table 2. Significant differences in slopes, intercepts, and QT60 were observed between the LQTS and control groups. Although no significant differences in the QT/HR slope were noted between the LQT3 and non‐LQT3 subgroups during exercise, pronounced differences in slopes and intercepts were observed during the face immersion. Representative examples of the QT/HR scatter diagrams for each phase of exercise and CWFI testing in each group are illustrated in Figure 1, demonstrating the linear relationship of the QT intervals with HR for each phase. Different patterns of QT dynamics were observed between LQT3 and non‐LQT3 subgroups during the CWFI tests. Figure 2 more clearly shows the difference in QT/HR slopes between LQT3 and non‐LQT3 subgroups during the CWFI tests. The QT/HR slopes were significantly steeper in both the LQT3 and non‐LQT3 subgroups than in the control group during exercise testing. This difference became more pronounced for the LQT3 subgroup during CWFI testing (Table 2). Schematic representation of the differential QT/HR relationship during exercise and CWFI tests is given in Figure 3.

**Table 2:** Linear regression analysis

QT dynamics	Control	LQTS	Subtypes	
		Total	Type 3	Non‐type 3
Slope				
Exercise	−1.21 ± 0.28	−2.16 ± 0.63**	−2.05 ± 0.57	−2.32 ± 0.73
Recovery	−1.13 ± 0.25	−1.98 ± 0.55**	−1.94 ± 0.56	−2.03 ± 0.57
Face immersion	−0.75 ± 0.24	−2.02 ± 0.76**	−2.42 ± 0.52**	−1.40 ± 0.65
Intercept				
Exercise	461 ± 42	589 ± 71**	578 ± 61	601 ± 87
Recovery	446 ± 34	562 ± 64**	556 ± 63	570 ± 69
Face immersion	427 ± 34	587 ± 71**	620 ± 55**	536 ± 63

Values are means ± SD where indicated.

HR, heart rate; LQTS, long QT syndrome; QT60, estimated QT interval at HR of 60 bpm calculated by the regression line formula.

*P < 0.05, **P < 0.01.

## Discussion

### Main findings

The present study demonstrated that the unadjusted QT intervals changed in a linear manner during the CWFI and exercise stress tests, which were carried out as described elsewhere.^21^ Notably, the QT/HR slope differed between the LQT3 and non‐LQT3 subgroups during the CWFI test, but not during the exercise test.

### QT dynamics for LQT3 during post‐exercise CWFI testing

Both clinical^25^ and experimental studies^11^ have revealed the differential responses of LQT1‐3 to beta‐adrenergic stimulation. Paradoxical QT interval prolongation during epinephrine and exercise stress testing can be used to differentiate LQT1 or 2 from healthy participants; however, LQT3 shows little response to stimulation of the sympathetic nervous system. The epinephrine stress test, in particular, may fail to distinguish LQT3 because of a supernormal QT shortening in response to such stimulation.^13^, ^15^ We previously reported that an analysis of QT dynamics using the unadjusted QT/HR slope during exercise testing can be used to differentiate patients with LQT3 from controls. However, this method is unable to differentiate LQT3 from other LQTS subtypes.^20^ Hekkala *et al.* reported that T‐wave morphology after an epinephrine bolus may be able to identify LQT3.^26^ The difficulty in reaching a definitive diagnosis means that there is a paucity of information regarding the QT dynamics of LQT3.^13^, ^17^, ^18^, ^19^ There are conceivably several possible explanations for discrepancies in the literature regarding this topic: first, HRs differ among stress test protocols (low‐dose/bolus epinephrine challenge, treadmill/bicycle exercise, etc.), and HRs are typically higher during an exercise test than during the epinephrine test. The different HRs obtained may indicate different sympathetic provocation levels. Second, autonomic changes during the exercise stress seem to be more complex than those during other protocols. In addition to a withdrawal of sympathetic tone, an accentuation of parasympathetic tone may also be noted during the recovery phase. This may explain why the measurement of the maximal QT interval following sympathetic stimulation has limitations in the identification of LQT (because of the various autonomic conditions). One previous report described the diagnostic utility of recovery phase QTc during treadmill exercise stress testing for the evaluation of LQTS, regardless of the subtype.^16^ Thus, it may be necessary to focus on the parasympathetic tone. In addition, we should adopt a different approach to investigating repolarization abnormalities, other than QT intervals themselves. In fact, a recent report focused on the non‐QTc ECG profile, including T‐wave morphology, as well as the QT interval.^27^

### Autonomic activity during post‐exercise CWFI testing

Several reports on post‐exercise autonomic activity have suggested that sympathetic withdrawal and parasympathetic activation occur at early stages of recovery.^17^, ^28^, ^29^, ^30^, ^31^ Previous studies have also revealed that parasympathetic activity is augmented during CWFI testing. Cold‐water face immersion, per se, elicits cardiac parasympathetic activity.^28^ Additionally, breath holding during CWFI induces vagal nerve stimulation. Thus, autonomic activity during post‐exercise CWFI is much more complex than that in other testing protocols. We were unable to evaluate the parasympathetic activity during CWFI using frequency‐domain analysis because of the limited number of participants (not shown). Clear HR lowering during post‐exercise CWFI testing may indicate that parasympathetic reactivation is also augmented during this test. Our finding that the abnormal QT response to HR changes during testing is concordant with the clinical characteristics of patients with LQT3, but not patients with LQT1 or 2, as patients with LQT3 often experience cardiac events when at rest or during sleep (at times in which the sympathetic tone is expected to be low).^32^ There are discrepancies in the QT/HR slope during CWFI between the present study and the previous study by Yoshinaga *et al.* ^24^ The specific reason for this remains unclear; however, the discrepancy might be attributable to the post‐exercise condition in the present study.

### Practical implications

Genetic diagnosing is becoming increasingly available and rapid. However, exercise testing in patients with LQTS may be able to provide valuable information, similar to that obtained from genetic testing. While LQT can be a concealed disease,^14^, ^33^ a simple stress test has the potential to unmask concealed forms of LQTS (LQT1, LQT2, and LQT3). Crotti *et al.* ^17^ confirmed that QTc prolongation during early recovery is the best parameter for predicting the LQT1 genotype, and patients with LQT2 are more likely to exhibit QT prolongation at the late phase of exercise recovery. The present study results showed that parasympathetic reactivation during post‐exercise CWFI can be used to predict LQT3 in patients with LQTS. Thus, provocative testing still has diagnostic value, particularly in guiding genetic testing for a definite diagnosis in borderline / concealed individuals. Furthermore, this testing could be used to validate the significance of genetic testing. Another way to recognize the phenomenon of excessive QT prolongation during bradycardia in patients with LQT3 is through the use of a Holter monitor to record the QT interval during nocturnal bradycardia.^34^ To the best of our knowledge, there is no previous report demonstrating that CWFI can predict cardiac events during swimming in patients with LQTS. However, there is one report indicating that the response to HR changes may be useful in identification of high‐risk patients with LQT1.^35^

Bennett *et al.* stated that in the case of LQT1, QT shortening in patients with higher HR on beta‐blocker therapy may be a therapeutic target for LQT management.^36^ Analogous to this, QT prolongation (dynamics) in slower HRs during post‐exercise CWFI may be a prognostic or therapeutic target for LQT3 management. Our data highlights the diagnostic value of provocative testing with CWFI in LQT3. However, testing this hypothesis clearly requires appropriate clinical trials. Patients with LQT3 may show a “silent” response to sympathetic stimulation (such as exercise), but can be at risk for the development of Torsade de Pointes ventricular tachycardia under triggering conditions, such as parasympathetic‐stimulating activities like swimming.

The likelihood of running and swimming is higher in children than in adults; we therefore speculate that the management of LQTS, particularly in children, can be investigated by using treadmill and post‐exercise CWFI tests, owing to the simple and non‐invasive nature of these methods.

### Cost effectiveness

Analysis of QT dynamics has the potential to be an alternative to drug‐challenge testing, as a non‐invasive assessment of ventricular repolarization abnormalities.^37^, ^38^ The present findings support the use of routine exercise stress testing in combination with post‐exercise CWFI, as it has diagnostic value and is readily available, safe, and non‐pharmacological in nature. Such ECG screening may decrease the total costs by the appropriate selection of candidates for genetic testing.

### Limitations of the study

This is a small study with several limitations that are common to those described in our previous studies. Our institute is a regional referral center, but the study population was derived from the school‐based ECG screening program. Therefore, patients may be biased toward having relatively low risk. Another limitation is that all patients with LQT3 had the same E1784K mutation, which may have a milder phenotype and different functional effects than those in some patients with other LQT3 mutations. The response to CWFI could be mutation‐dependent. Another concern is the extremely heterogeneous and small group of “non‐LQT3” children (consisting of 8 children), which included subtypes 1, 2, 7, and 8. Hence, the study population was not sufficient to evaluate the prognosis. Another potential limitation is that genetic analyses were not performed in the controls. Finally, in the present study, the target duration of CWFI was 30 s or more. Although the CWFI duration reached at least 20 s in all participants, it did not reach the target (30 s) in some young participants. The minimal HR did not decrease to <50% of the maximal HR during the CWFI test in some participants. Thus, we must admit that a limitation of the CWFI test is an inadequate CWFI load, particularly, in smaller children.

## Conclusions

The present study showed that the evaluation of QT dynamics using fully automated QT measurement is possible during parasympathetic provocative tests, such as post‐exercise CWFI testing. Patients with LQT3 exhibited different QT dynamics during post‐exercise CWFI testing. Abnormal QT responses to HR changes during testing were concordant with the clinical characteristics of LQT3, in that cardiac events occur at low HRs or during sleep. These findings increase the chance of LQT3 detection. Larger studies are required to evaluate the diagnostic or prognostic value of LQTS.

## Disclosure

The authors declare no conflicts of interest.

## Author contribution

K.T. designed the study. K.T., N.M., W.S., and M.N. collected the data. K.T. wrote the manuscript. N.M, and W.S. gave technical support and conceptual advice. K.T. performed the statistical analysis and drafted the manuscript. W.S. critically reviewed the manuscript. All authors read and approved the final manuscript.

## Supporting information


**Table S1.** Gene analysis for the all subjects with LQTS.

## Data Availability

The deidentified participant data will not be shared.
