# Supplementary material for: Dynamic QT response to cold‐water face immersion in long‐QT syndrome type 3
Source: Pediatr Int. 2020 Aug 6;62(8):899–906. doi: 10.1111/ped.14319 (PMC7496693; doi:10.1111/ped.14319)
Supplement: Supplementary file 1 — Table S1. Gene analysis for the all subjects with LQTS. [file PED-62-899-s001.docx]

**SUPPLEMENTAL MATERIALS**

I. Supplemental tables

Table S1: Gene analysis for the all subjects with LQTS

|  | Genotypes | Sex | Years at testing | Genes | Nucleotide | Amino acid | Types of mutations | Location |
| --- | --- | --- | --- | --- | --- | --- | --- | --- |
| 1 | LQT 3 | M | 8 | *SCN5A* | c.5353 G>A | p.E1784K | missense | C-terminus |
| 2 | LQT 3 | M | 10 | *SCN5A* | c.5353 G>A | p.E1784K | missense | C-terminus |
| 3 | LQT 3 | F | 9 | *SCN5A* | c.5353 G>A | p.E1784K | missense | C-terminus |
| 4 | LQT 3 | F | 12 | *SCN5A* | c.5353 G>A | p.E1784K | missense | C-terminus |
| 5 | LQT 3 | M | 10 | *SCN5A* | c.5353 G>A | p.E1784K | missense | C-terminus |
| 6 | LQT 3 | M | 16 | *SCN5A* | c.5353 G>A | p.E1784K | missense | C-terminus |
| 7 | LQT 3 | M | 12 | *SCN5A* | c.5353 G>A | p.E1784K | missense | C-terminus |
| 8 | LQT 3 | M | 7 | *SCN5A* | c.5353 G>A | p.E1784K | missense | C-terminus |
| 9 | LQT 3 | M | 15 | *SCN5A* | c.5353 G>A | p.E1784K | missense | C-terminus |
| 10 | LQT 3 | M | 7 | *SCN5A* | c.5353 G>A | p.E1784K | missense | C-terminus |
| 11 | LQT 3 | F | 6 | *SCN5A* | c.5353 G>A | p.E1784K | missense | C-terminus |
| 12 | LQT 3 | F | 8 | *SCN5A* | c.5353 G>A | p.E1784K | missense | C-terminus |
| 13 | LQT1 | F | 6 | *KCNQ1* | c.1084_1085 ins GGCAGA | p.Q361_K362 ins RQ | in-frame insertion | C-terminus |
| 14 | LQT1 | F | 8 | *KCNQ1* | c.1084_1085 ins GGCAGA | p.Q361_K362 ins RQ | in-frame insertion | C-terminus |
| 15 | LQT1 | F | 7 | *KCNQ1* | c.1663 C>T | p.R555C | missense | C-terminus |
| 16 | LQT2 | F | 19 | *KCNH2* | c.1681G>A | p.A561T | missense | S5 |
| 17 | LQT2 | F | 12 | *KCNH2* | c.1260_1261 ins CATCTAC | p.T421fsX520 | frameshift insertion | S1 |
| 18 | LQT2 | F | 9 | *KCNH2* | c.1260_1261 ins CATCTAC | p.T421fsX520 | frameshift insertion | S1 |
| 19 | LQT7 | F | 13 | *KCNJ2* | c.22 C>T | p.R8C | missense | N-terminus |
| 20 | LQT8 | M | 14 | *CACNA1C* | c.3343G>A | p.E1115K | missense | D3/S5-S6 |
